# Type 1 diacylglycerol acyltransferases of *Brassica napus* preferentially incorporate oleic acid into triacylglycerol

**DOI:** 10.1093/jxb/erv363

**Published:** 2015-07-20

**Authors:** Jose Aznar-Moreno, Peter Denolf, Katrien Van Audenhove, Stefanie De Bodt, Steven Engelen, Deirdre Fahy, James G. Wallis, John Browse

**Affiliations:** ^1^Institute of Biological Chemistry, Washington State University, Pullman, WA 99164-6340, USA; ^2^Bayer CropScience N.V., Technologiepark 38, B-9052 Ghent, Belgium

**Keywords:** DGAT1, fatty acids, seed lipid metabolism, seed oil, triacylglycerol, transcript profile.

## Abstract

DGAT1 enzymes (acyl-CoA:*d*iacyl*g*lycerol *a*cyl*t*ransferase 1, EC 2.3.1.20) catalyse the formation of triacylglycerols (TAGs), the most abundant lipids in vegetable oils. Thorough understanding of the enzymology of oil accumulation is critical to the goal of modifying oilseeds for improved vegetable oil production. Four isoforms of BnDGAT1, the final and rate-limiting step in triacylglycerol synthesis, were characterized from *Brassica napus*, one of the world’s most important oilseed crops. Transcriptional profiling of developing *B. napus* seeds indicated two genes, *BnDGAT1-1* and *BnDGAT1-2*, with high expression and two, *BnDGAT1-3* and *BnDGAT1-4*, with low expression. The activities of each BnDGAT1 isozyme were characterized following expression in a strain of yeast deficient in TAG synthesis. TAG from *B. napus* seeds contain only 10% palmitic acid (16:0) at the *sn*-3 position, so it was surprising that all four BnDGAT1 isozymes exhibited strong (4- to 7-fold) specificity for 16:0 over oleic acid (18:1) as the acyl-CoA substrate. However, the ratio of 18:1-CoA to 16:0-CoA in *B. napus* seeds during the peak period of TAG synthesis is 3:1. When substrate selectivity assays were conducted with 18:1-CoA and 16:0-CoA in a 3:1 ratio, the four isozymes incorporated 18:1 in amounts 2- to 5-fold higher than 16:0. This strong sensitivity of the BnDGAT1 isozymes to the relative concentrations of acyl-CoA substrates substantially explains the observed fatty acid composition of *B. napus* seed oil. Understanding these enzymes that are critical for triacylglycerol synthesis will facilitate genetic and biotechnological manipulations to improve this oilseed crop.

## Highlight

Fatty acid composition determines oil qualities. Not only the selectivity of BnDGAT1 enzymes, but also the concentration of the fatty acid substrates, determines the oil composition in *Brassica napus* seeds.

## Introduction

Acyl-CoA:diacylglycerol acyltransferase enzymes (DGAT; EC 2.3.1.20) transfer a fatty acid from CoA to the *sn-3* position of a diacylglycerol (DAG) molecule, producing a triacylglycerol (TAG), which is the principal storage lipid in plant seeds, as well as in animals and yeast (Bates and Browse, 2012; Yen *et al.*, 2008). DGAT enzymes catalyse the final, committed step of TAG synthesis, and probably represent the rate-limiting step of oil synthesis. DGAT enzymes affect not only the quantity of oil produced, but also the quality and fatty acid composition of oils through their substrate preferences (Cahoon *et al.*, 2007).

DGAT enzymes have been much examined with a view to manipulating oil biochemistry, with the potential to satisfy increasing demands for vegetable oil production. Vegetable oils are in such demand because they are an important component of human diet (FAO expert consultation, 2010) and also because they are useful for fuel production (Andrianov *et al.*, 2010) and as feedstocks for chemical industries (Carlsson *et al.*, 2011). Modification of oil quality is particularly important for human health: for example, oleic acid, a monounsaturated fatty acid, offers considerable health and cooking benefits as a component of dietary oil, especially when compared to saturated and polyunsaturated fatty acids (Gillingham *et al.*, 2011).

The acyl-CoA:diacylglycerol acyltransferase reaction is facilitated by two families of proteins that are not closely related. A DGAT2 family supports the major flux of TAG synthesis in mammals and yeast (Yen *et al.*, 2008), while in plants members of a separate DGAT1 family are determinative for seed TAG synthesis in *Brassica napus, Arabidopsis* and many other oilseeds. Several lines of evidence support this conclusion, including antisense suppression of DGAT1 in *B. napus* that reduced seed oil content by nearly 30% (Lock *et al.*, 2009) and analysis of a mutation in the DGAT1 of *Arabidopsis* that decreased seed lipids by 45% (Katavic *et al.*, 1995; Routaboul *et al.*, 1999). Further, overexpression of DGAT1 increases seed oil content in *B. napus* (Taylor *et al.*, 2009; Weselake *et al.*, 2008). In plants, the activity of the DGAT2 family of enzymes is most often associated with incorporation of less common forms of fatty acids into TAG, for example ricinoleic acid in castor, *Ricinus communis*, (Burgal *et al.*, 2008) or eleostearic acid in tung tree, *Vernicia fordii*, (Shockey *et al.*, 2006).

The biochemical activities of DGAT enzymes have been routinely assayed by isolating microsomal fractions of yeast strains engineered for heterologous expression of the enzymes, followed by *in vitro* assays with appropriate DGAT substrates. Substrate preferences of DGAT in different plants have been studied with respect to specificity and selectivity, with assays using as substrates either a single acyl-CoA species or a combination of acyl-CoA substrates in equimolar mixture (Lung and Weselake, 2006). It is clear that DGAT enzymes not only play a decisive role in the specific channelling of fatty acids into TAG molecules (Zhang *et al.*, 2009), but are also influenced by other factors such as availability of specific diacylglycerol substrates and the composition of the acyl-CoA substrate pool available to the enzyme (Shockey *et al.*, 2006).

In this report, four cloned DGAT1-family enzymes from the oilseed crop *B. napus* are characterized by expression in yeast, comparing their activities by *in vitro* activity assays using microsomal preparations from yeast cultures that express the DGAT genes. The selectivity and specificity of the enzymes are determined for appropriate DAG and acyl-CoA substrates, including a determination based on the ratio of acyl-CoA levels found in *B. napus* seeds. With an *in vivo* assay, changes in the fatty acid composition of TAG are also characterized when yeast expressing these DGAT1 enzymes are cultured in medium supplemented with palmitic or oleic acid.

## Materials and methods

### *Cloning of four* Brassica napus *DGAT1 cDNAs*

Sequences homologous to known DGAT enzymes were retrieved from a database of cDNA sequences derived from *B. napus* developing seed using the BLASTP program (www.ncbi.nlm.nih.gov; Altschul *et al.*, 1990) The four candidate BnDGAT isoforms were amplified from the library using primers designed to facilitate cloning of the PCR products into pMK195 (Overvoorde *et al.*, 1996). This yeast vector provides constitutive expression of the inserted open reading frames using the ADH1 promoter. The four resulting constructs were confirmed by DNA sequencing and then separately transformed, along with the empty vector as control, into a quadruple mutant of yeast, H1246 [*are1*, *are2* (acyl-CoA:sterol acyltransferase 1 and 2), *lro1* (lecithin cholesterol acyl transferase), *dga1* (diacylglycerol O-acyltransferase)], which is devoid of TAG synthesis (Sandager *et al.*, 2002). The wild-type yeast strain used was *Saccharomyces cerevisiae* SCY62, the parent of H1246 (Sandager *et al.*, 2002).

### Sequence analysis

Sequences homologous to the candidate BnDGAT enzymes were retrieved using BLASTP. Amino acid sequences were aligned with candidate BnDGAT sequences using ClustalX v.2.0.10 under the default settings (Larkin *et al.*, 2007), and the alignments used to generate a phylogenetic tree with a neighbor-joining algorithm (Saitou and Nei, 1987); the final phenogram was created with MEGA 5.0 (Tamura *et al.*, 2011). The bioinformatics program TMHMM (http://www.cbs.dtu.dk/services/TMHMM-2.0/) was used to predict transmembrane regions. Sequences corresponding to BnDGAT1 found in public databases include BnDGAT1-1; JN224474, BnDGAT1-2; JN224473, BnDGAT1-3; JN224476, and BnDGAT1-4; JN224475. Systematic names for the BnDGAT1 genes are BnDGAT1-1; BnADGAT1.a, BnDGAT1-2; BnCDGAT1.a, BnDGAT1-3; BnCDGAT1.b, and BnDGAT1-4; BnADGAT1.b.

### Yeast expression and microsome preparation

The yeast mutant H1246 was transformed with constructs expressing the BnDGAT candidates followed by selection on solid medium lacking uracil, the prototrophic selection marker for pMK195. Single colonies were inoculated into liquid medium and cultured for 2 d at 30°C in a shaker at 220rpm. Cultures were diluted to an OD_600_ of 0.05, incubated overnight at 30°C, and microsomes prepared as described in Zheng *et al.* (2008) with minor modification. Yeast cells were harvested by centrifugation, the pellets washed with water then resuspended in 2ml lysis buffer [20mM Tris-HCl pH 7.9, 10mM MgCl_2_, 1mM EDTA, 1mM DTT, 0.3M (NH_4_)_2_SO_4_, 5% glycerol]. Two millilitres of glass beads (Sigma) were added and cells lysed by five repetitions of 30 s vortexing, 30 s on ice. An additional 1ml of lysis buffer was added before the sample was centrifuged at 10 000 ×*g* for 30 m, after which the upper phase was removed and centrifuged at 100 000 ×*g* for 1h. The microsomal pellet was resuspended in 400 µl of 100mM KH_2_PO_4_/K_2_HPO_4_ buffer (pH 7.2) and aliquots frozen in liquid nitrogen before storage at -80°C. Protein concentration was determined using the Bradford method conducted with Bio-Rad protein reagent (Bio-Rad, Hercules, CA, USA) with bovine serum albumin as the standard.

### Embryo transcriptional profiling

Transcriptional profiling of the four BnDGAT1 genes from four developmental stages of the embryo was conducted as described (Troncoso-Ponce *et al.*, 2011). Briefly, the seeds of greenhouse-grown *B. napus* were collected at intervals of 18–20, 23–25, 28–30, and 33–35 days after flowering (DAF) and frozen. After collection, total RNA was extracted using TRIZOL (Invitrogen, http://www.invitrogen.com/), followed first by mRNA purification with a commercial kit (Illustra, GE Healthcare, http://www.gehealthcare.com/) then by conversion to cDNA with Superscript III (Life Technologies, http://www.lifetechnologies.com/us/en/home.html) according to the manufacturer’s instructions. For sequence analysis, cDNA was processed with a Roche Library Preparation Kit (http://www.roche.com/) and sequenced with a 454 sequencer (GS FLX, Roche). Expression of the gene family was quantified taking into account the similarity between the gene sequences. For a given gene, the repartitioning of reads is based on the ratio of the number of reads mapped uniquely on this gene to the total number of reads mapped uniquely on the genes sharing the common reads. FPKM = number of reads × (1000/gene length) × (1 million/total number of reads).

### Assay of diacylglycerol acyltransferase activity

Assays were conducted in microfuge tubes at 30°C in a shaking water bath, with a total volume of 100 µl, by adding microsome extract containing 20 µg of protein to initiate the reaction. The standard reaction mixture consisted of 50mM KH_2_PO_4_/K_2_HPO_4_ buffer pH 7.2, 1mg ml^-1^ BSA, 320 µM DAG (1-palmitoyl-2-oleoyl-*sn*-glycerol or 1,2-dioleoyl-*sn*-glycerol), with [1-^14^C]-labelled palmitoyl-CoA or oleoyl-CoA at 60 mCi/mmol^-1^, or 1-^3^H-labelled oleoyl-CoA at 60 Ci mmol^-1^) (Lung and Weselake, 2006). Enzyme reactions were allowed to proceed for 5min, then terminated by adding of 10 µl of 5% (w/v) SDS. Lipids were extracted with 2ml of hexane:isopropanol (3:2) and the upper organic phase transferred to a fresh tube and dried under nitrogen gas. The lipids were resolubilized in 100 µl chloroform and applied to TLC plates (Whatman Partisil K6, http://www.whatman.com) which were developed in 80:20:1 (V/V) hexane:diethylether:acetic acid. TAGs were visualized and marked by staining in iodine vapour. Relevant portions of silica were scraped from the plates, combined with 5ml of liquid scintillant and analysed using a scintillation counter (Packard, Tri-Carb 2100TR). Enzyme assays were performed in triplicate using yeast transformed with the empty vector as control.

### Fatty acid and TAG analysis

For fatty acid analysis, 5ml yeast pre-cultures were grown for 48h at 30°C. One ml of each culture was transferred to 100ml of medium supplemented with 0.01% Tween 80 and fatty acid supplements where indicated, to a final concentration of 500 µM. After 24h shaking incubation at 30°C, cells were harvested by centrifugation, washed with 20ml of 0.1M NaHCO_3_, and lysed by vortexing with 2ml of glass beads in 5ml chloroform:methanol (2:1), with five repetitions of 30 s vortexing, 30 s on ice. The lipids were extracted from the lysate twice by shaking for 2h with 15ml of chloroform:methanol (2:1) (Domergue *et al.*, 2003). The resulting organic phases were combined, then extracted with 15ml of 0.5% NaCl, dried with Na_2_SO_4_ and evaporated under nitrogen gas. The residue was dissolved in 2ml of chloroform and purified by thin layer chromatography using chloroform:methanol:acetic acid (80:20:1) as solvent. The TAG fraction was visualized under iodine vapour, and for total TAG determination scanned and analysed with ImageJ software, comparing with a standard (1-palmitoyl-2-oleoyl-3-linoleoyl-rac-glycerol) whose concentration was known. Otherwise, the portion of the plate containing TAG fractions was scraped from it and fatty acid compositions analysed by preparation of fatty acid methyl esters and by gas chromatographic analysis as described in Miquel and Browse (1992).

## Results

### Transcript profiling of four *Brassica napus* DGAT1 genes

**Fig. 1.**
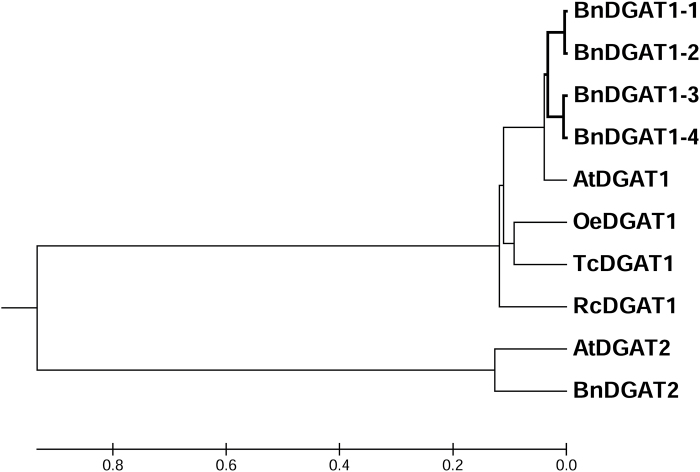
Phylogenetic relationships among four *B. napus* DGAT1 proteins and other plant diacylglycerol acyltransferases. Plant species included in the phylogenetic tree are: At, *Arabidopsis thaliana*; Bn, *Brassica napus*; Oe, *Olea europaea*; Tc, *Theobroma cacao*; Rc, *Ricinus communis*. BnDGAT1 proteins are also referred to as BnDGAT1-1 (BnADGAT1.a), BnDGAT1-2 (BnCDGAT1.a), BnDGAT1-3 (BnCDGAT1.b), BnDGAT1-4 (BnADGAT1.b) (Greer *et al.*, 2014).

Protein sequences predicted from transcriptome analysis of *B. napus* developing seed were analysed for similarity with known plant DGAT1 proteins, using the BLASTP algorithm (Altschul *et al.*, 1990). Four candidate DGAT sequences were identified for further analysis. The evolutionary relationships within this candidate *B. napus* DGAT family were analysed by performing a phylogenetic analysis with a set of protein sequences from diverse organisms (Fig. 1). The four DGAT1-like sequences clustered within the DGAT1 family and are strongly related to the well-characterized *Arabidopsis* DGAT1, with ∼85% amino acid sequence identity. The four predicted enzymes were designated as BnDGAT1-1 through BnDGAT1-4; the same four have recently been named by others (Fig. 1; Greer *et al.*, 2014).

**Fig. 2.**
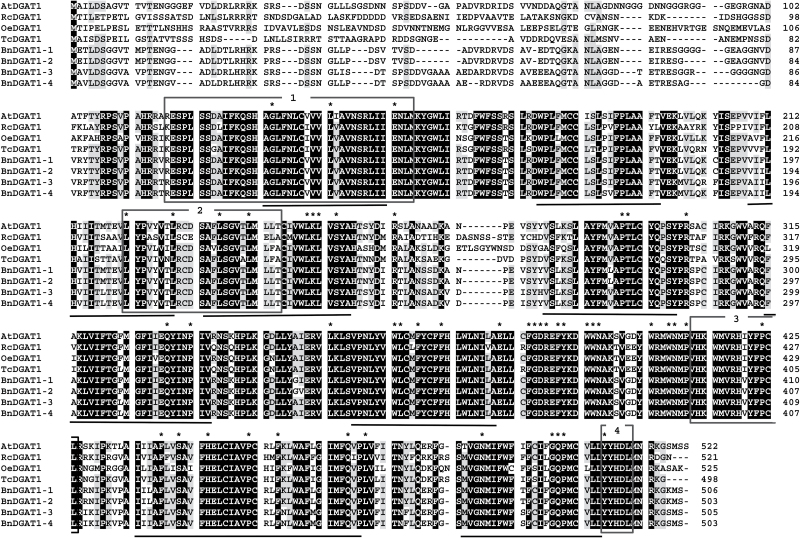
Sequence alignment of deduced amino acid sequences of four *B. napus* DGAT1 candidates (BnDGAT1-1 to BnDGAT1-4) and DGAT1 proteins from *Arabidopsis thaliana* (At), *Ricinus communis* (Rc), *Olea europaea* (Oe) and *Theobroma cacao* (Tc). Nine predicted transmembrane domains are underlined. Boxed regions are (1) acyl-CoA binding or active site, (2) thiolase acyl-enzyme binding signature, (3) DAG binding site and (4) ER retention/retrieval. Black and grey backgrounds indicate 100% and 75% conservation, respectively, among the eight sequences shown. Asterisks indicate residues conserved more widely among DGAT1 proteins (refer to text for details).

Within the BnDGAT family, BnDGAT1-1 and BnDGAT1-2 are 98% identical to each other in primary amino acid sequence, and BnDGAT1-3 and 1-4 likewise 97% identical. However, amino acid comparisons between these two groups show lower, ~88%, identity. Alignment of the deduced amino acid sequences of these enzymes with *Arabidopsis* and other plant DGAT1 sequences (Fig. 2) revealed a strong homology, which does not, however, include the first 90 amino acid residues; these first residues are known to be variable within the DGAT1 family (Turchetto-Zolet *et al.*, 2011). The DGAT1 variable N-terminal region has been shown to allow the proteins to form homo-tetramers (McFie *et al.*, 2010). The candidate DGAT1 proteins are predicted to include the nine putative transmembrane domains and a C-terminal ER retrieval motif characteristic of DGAT1 enzymes (Fig. 2; Turchetto-Zolet *et al.*, 2011). Each of the proteins shares functional motifs present in the DGAT1 family, including an acyl-CoA binding and active site (Fig. 2, box 1), a DAG-binding site (Fig. 2, box 3), and a putative thiolase acyl-enzyme binding signature which includes a leucine zipper motif (Fig. 2, box 2; Cao, 2011).

**Fig. 3.**
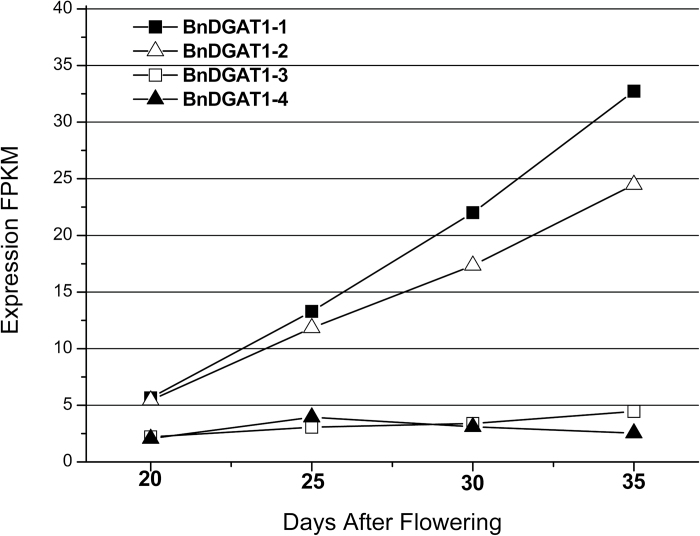
Transcriptional profiling *BnDGAT1*. The transcripts from four candidate *BnDGAT1* genes are shown at four stages of seed development. Data are represented as fragments per kilobase of transcript per million mapped reads (FPKM) = number of reads × (1000/gene length) × (1 million/total number of reads). (Refer to text for details.)

Transcriptional profiles of developing *B. napus* seeds were analysed to determine expression of the *BnDGAT1* genes through the course of seed development. Two genes, *BnDGAT1-1* and *BnDGAT1-2*, exhibited moderate levels of transcript, ∼5 *f*ragments *p*er *k*ilobase of transcript per *m*illion mapped reads (FPKM), early in seed development, after which the transcript for both rapidly increased 5- to 6-fold over the course of seed development, reaching 25−35 FPKM by 33−35 days after flowering (DAF) (Fig. 3). This transcript profile accords with the reported developmental profile of DGAT enzyme activity assayed in developing *B. napus,* when activity peaked at 35 DAF (Weselake *et al.*, 1993). Expression of RNA for the other two isoforms, *BnDGAT1-3* and *BnDGAT1-4,* was consistently much lower, starting near 2.5 FPKM for the first (20 DAF) interval and not exceeding 5 FPKM during the entire developmental course (Fig. 3).

### Acyl-CoA specificity of BnDGAT1 isozymes

**Fig. 4.**
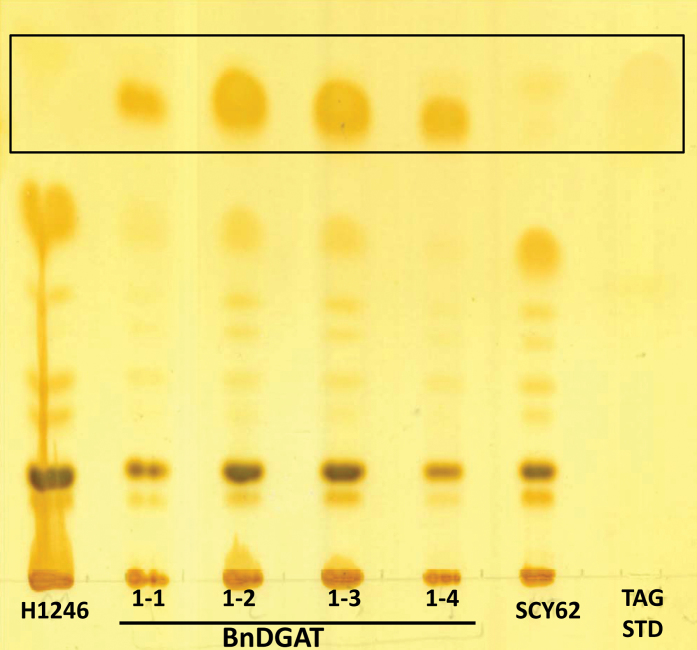
Separation of yeast lipids by TLC. Expression of each BnDGAT1 recovers TAG synthesis in yeast H1246, a TAG synthesis quadruple mutant (*are1*, *are2*, *lro1*, *dga1*). The mutant transformed with the empty vector served as negative control. The wild type is SCY62, and the right lane is the TAG standard. Triacylglycerols are boxed.

To confirm the functionality of the four candidate enzymes, each of them were expressed in the quadruple mutant yeast strain H1246, mutated in all four TAG synthesis genes (*are1*, *are2*, *lro1*, and *dga1*), so that the strain synthesizes no TAG (Sandager *et al.*, 2002). When expressed in this yeast mutant strain, each of the *B. napus* enzymes supported TAG synthesis (Fig. 4). These results encouraged the authors to proceed with a more detailed functional analysis of enzyme activities by assaying microsomal preparations from the mutant yeast expressing each of the four *B. napus* DGAT1 isoforms. For these assays, one of two DAG substrates were supplied, either palmitoyl/oleoyl DAG (1-palmitoyl-2-oleoyl-sn-glycerol, PO-DAG) or oleoyl/oleoyl DAG (1,2-dioleoyl-sn-glycerol, OO-DAG), appropriate physiological DAG substrates for TAG synthesis in *B. napus* seeds. For fatty acid substrates, [^14^C]-labelled palmitoyl-CoA (16:0-CoA) and [^14^C]-labelled oleoyl-CoA (18:1-CoA) were provided.

**Fig. 5.**
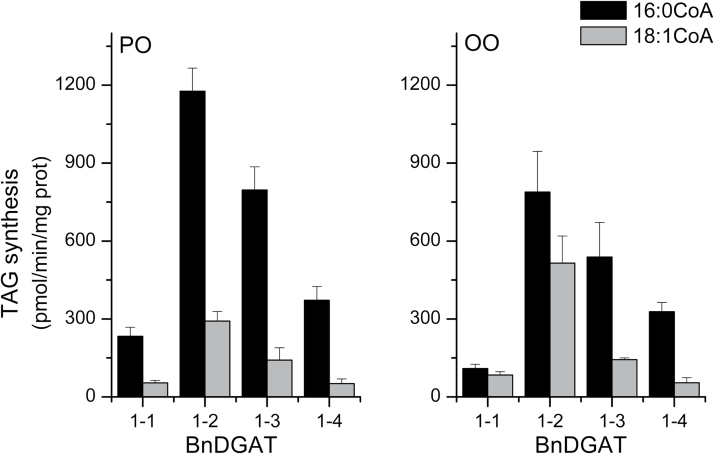
Specificity of BnDGAT1 isozymes. Microsome aliquots from the yeast TAG mutant H1246 expressing the indicated BnDGAT1 isozyme were supplied with [^14^C]-palmitoyl-CoA or [^14^C]-oleoyl-CoA, along with either 1-palmitoyl-2-oleoyl-sn-glycerol (PO-DAG) or dioleoyl-*sn*-glycerol (OO-DAG). The DGAT1 activity was calculated based on radioactivity incorporated into TAG; results are means ±SD, *n*=3.

Specificity of the DGAT enzymes for 16:0 and 18:1 were first examined by supplying the acyl-CoAs at 15 µM in separate reactions. All the BnDGAT1 isozymes were strongly specific for incorporation of palmitate (16:0) into PO-DAG, exhibiting both different levels of incorporation and different degrees of selective preference for 16:0 over 18:1. While interpretation of the absolute level of incorporation for each enzyme must be qualified by the vagaries of heterologous expression and *in vitro* analysis, the specificity of the enzymes for the substrates shows that, using PO-DAG, BnDGAT1-4 demonstrated the strongest preference for 16:0 over 18:1 incorporation, followed by BnDGAT1-1, BnDGAT1-3, and finally BnDGAT1-2 as the least specific. When using OO-DAG, BnDGAT1-4 again exhibited strong specificity for 16:0, but the other isozymes incorporated a lower ratio of 16:0 to 18:1 because 16:0 incorporation was lower than with PO-DAG cosubstrate (Fig. 5). In this heterologous expression system, the most active DGAT1 isoforms were BnDGAT1-2 and BnDGAT1-3.

### Acyl-CoA selectivity of the BnDGAT1 isozymes

**Fig. 6.**
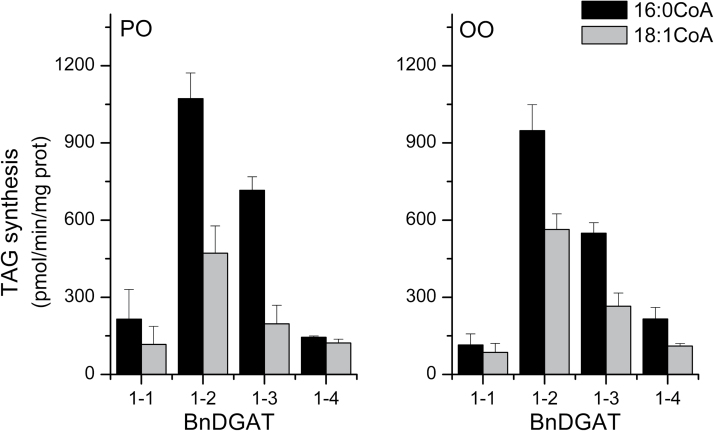
Selectivity of BnDGAT1 isozymes with equimolar acyl-CoA substrates. Microsome aliquots from the yeast TAG mutant H1246 expressing the indicated BnDGAT1 isozyme were supplied with [^14^C]-palmitoyl-CoA and simultaneously with [^3^H]-oleoyl-CoA, along with either 1-palmitoyl-2-oleoyl-sn-glycerol (PO-DAG) or dioleoyl-*sn*-glycerol (OO-DAG). 15 µM of each acyl-CoA were supplied. The DGAT1 activity was calculated based on radioactivity incorporated into TAG; results are means ±SD, *n*=3.

Next, selectivity of the four isozymes for the two principal acyl-CoA substrates were examined by conducting assays including both 16:0-CoA and 18:1-CoA together in the assays, as [^14^C]-labelled palmitoyl-CoA and [^3^H]-labelled oleoyl-CoA. When activities in the presence of equimolar mixtures of the substrates were compared (Fig. 6), the results indicated that 16:0-CoA was again the preferred substrate; BnDGAT1-3 showed the highest selectivity for incorporating 16:0 with either PO-DAG or OO-DAG substrate, followed by BnDGAT1-1, BnDGAT1-2, and finally BnDGAT1-4. BnDGAT1-4 was notably less active in incorporating 16:0 into TAG when the 18:1 substrate is also present, losing ∼40% of its 16:0-incorporation activity. The four enzymes all incorporated more oleoyl fatty acid than when assayed separately, but the changes were quite modest (Fig. 6).

**Fig. 7.**
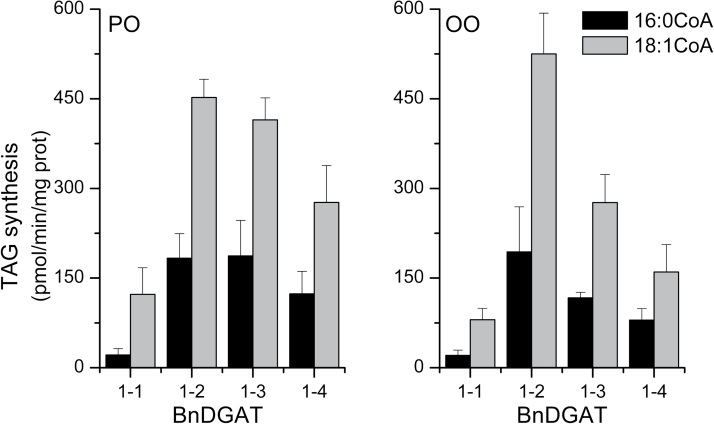
Selectivity BnDGAT1 isozymes with low palmitoyl-CoA concentration. Microsome aliquots from the yeast TAG mutant H1246 expressing the indicated BnDGAT1 isozyme were supplied with [^14^C]-palmitoyl-CoA simultaneously with [^3^H]-oleoyl-CoA, along with either 1-palmitoyl-2-oleoyl-sn-glycerol (PO-DAG) or dioleoyl-*sn*-glycerol (OO-DAG). 5µM palmitoyl-CoA and 15 µM oleoyl-CoA were supplied. The DGAT1 activity was calculated based on radioactivity incorporated into TAG; results are means ±SD, n=3.

While equimolar substrates are typically used in selectivity experiments (Lung and Weselake, 2006), a 1:3 ratio of palmitoyl-CoA to oleoyl-CoA is more representative of the acyl-CoA pool in *B. napus* between 30 and 40 days after pollination, when rapid deposition of storage TAG is taking place (Perry and Harwood, 1993; Larson *et al.*, 2002). Therefore additional experiments were conducted with the acyl-CoA substrates present at a 1:3 ratio, with 5 µM 16:0-CoA and 15 µM 18:1-CoA. The effect of approximating the physiological ratio of substrates was striking. All the enzymes incorporated 16:0 at much lower rates, while maintaining or increasing 18:1 incorporation using PO-DAG (Fig. 7). Incorporation of 18:1 with OO-DAG was essentially unchanged. It was questioned whether this difference was simply due to the lower 16:0 concentration, but when 5 µM 16:0 was provided as the sole acyl-CoA substrate, incorporation was comparable to the rates measured at 15 µM (Table 1).

**Table 1.** Activity of BnDGAT1 enzymes with 5 µM [^14^C]-Palmitoyl-CoA All results are means ±SD, *n*=2.

Enzyme	DAG substrate	
	PO	OO
BnDGAT1-1	202±43	190±13
BnDGAT1-2	978±32	819±42
BnDGAT1-3	907±20	538±24
BnDGAT1-4	676±60	466±17

### Triacylglycerol synthesis in yeast expressing BnDGAT1 isozymes

**Fig. 8.**
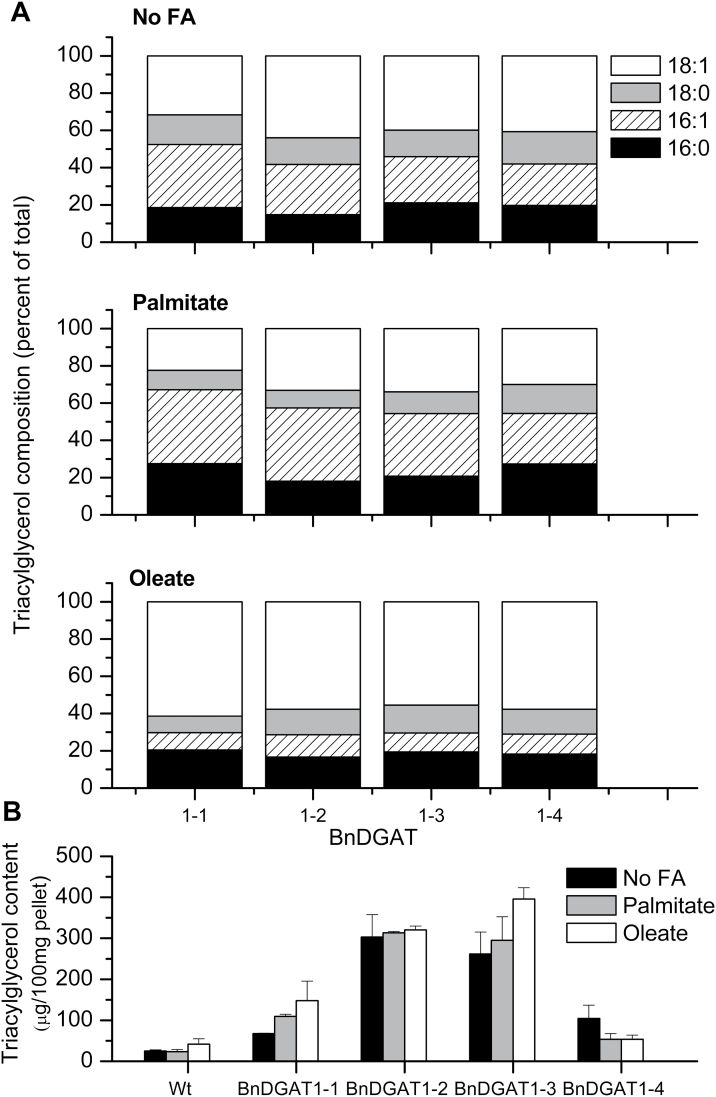
TAG synthesis by mutant yeast cultures expressing BnDGAT1 enzymes. Yeast were cultured either without fatty acid (No FA), or supplemented with palmitic acid (Palmitate) or with oleic acid (Oleate) to a 500 µM final concentration. Incubation was for 24h at 30°C. (A) Fatty acid composition of isolated TAG under each culture condition; results are means, *n*=3. (B) Total TAG produced by BnDGAT1 enzymes; results are means ±SD, *n*=3.

Properties of the BnDGAT1 enzymes *in vivo* were further examined by measuring TAG synthesis of H2146 yeast expressing the enzymes, first without added fatty acid in the medium, then by adding separately either palmitic or oleic acid. Since yeast take up these fatty acids from the medium by esterification to CoA (Faergeman *et al.*, 2001), the supplements provide excess acyl-CoA substrate to the expressed BnDGAT1 enzymes *in vivo*. After two days’ growth the total TAG accumulated was measured and the fatty acid composition of the TAG analysed. Total 16-carbon fatty acids are the sum of 16:0 (either the product of yeast fatty acid synthase or taken up from the media) and 16:1, the result of 16:0 desaturation by the yeast OLE1p ∆9 desaturase. The 18-carbon fatty acids are 18:0, synthesized by the yeast, and 18:1, whether taken up from the media or the product of 18:0 desaturation by OLE1p. In the absence of any fatty acid supplement, the fractions of fatty acid incorporated by each BnDGAT1 isoform were quite similar, with an average of 45% 16-carbon fatty acids (Fig. 8A). In cultures supplemented with palmitic acid, the proportion of 16-carbon fatty acids increased to ∼51% of total TAG; most of the increase came as 16:1, and the BnDGAT1-1 isozyme incorporated a higher proportion of 16:0+16:1 than the other BnDGAT1 forms. In contrast, oleic acid supplementation reduced 16-carbon fatty acids in TAG to <30% of the total, and 18:1 made up ∼60% of the total TAG fatty acids in yeast supplemented with oleate (Fig. 8A).

When total TAG content of H1246 yeast expressing each of the four different DGAT1 isozymes was determined, the TAG composition was increased, relative to the control wild-type strain (SCY62), by expression of each BnDGAT1 isozyme, even without fatty acid supplement (Fig. 8B). Addition of fatty acid supplements to the medium generally led to little or no increase in the accumulation of TAG. Both BnDGAT1-2 and BnDGAT1-3 were highly active in producing TAG in this heterologous expression system, producing 5- or 6-fold more TAG than wild-type yeast, while increases from BnDGAT1-1 and BnDGAT1-4 expression were ∼3-fold (Fig. 8B).

## Discussion

Research into the DGAT family of enzymes ranges from studies on their role in human health (Yen *et al.*, 2008) to manipulation of their expression in microalgae or fungi to develop improved biofuels (Liang and Jiang, 2013), but the present study focuses on the roles of the enzymes in oilseed TAG synthesis (Chapman and Ohlrogge, 2012). *Brassica* crops are the third most important edible oil crop, providing oil high in oleic acid, with direct benefits for human health as well as for thermostable cooking oil (Rahman, 2014). Because DGAT enzymes can exert strong flux control for TAG synthesis, they are promising targets for engineering oil seeds (Jako *et al.*, 2001; Cahoon *et al.*, 2007; Weselake *et al.*, 2008). In *B. napus* and *Arabidopsis*, the DGAT1 enzymes play a major role in seed lipid accumulation (Weselake *et al.*, 2008; Zhang *et al.*, 2009). Transcriptional data from *B. napus* developing seeds were examined, revealing four predicted protein sequences with similarity to DGAT1 enzymes from other organisms (Figs 1, 2; Jako *et al.*, 2001; He *et al.*, 2004). The topology of DGAT1 enzymes is not settled. The mammalian DGAT1 proteins contain nine predicted transmembrane domains (like the plant enzymes, Fig. 2), but topology analysis indicates that the protein may have as few as three membrane spans (McFie *et al.*, 2010), with the ends of the protein on opposite sides of the membrane. A different model based on inhibitor studies suggests that the protein may adopt dual membrane topology *in vivo* (Wurie *et al.*, 2011).

The candidate BnDGAT1 isozymes were characterized by expression in yeast strain H1246 in which TAG synthesis has been eliminated (Sandager *et al.*, 2002). Expression of each of the four BnDGAT1 enzymes restored TAG synthesis (Fig. 4). Having confirmed their enzymatic activity, transcript levels of the corresponding genes in developing *B. napus* seeds were examined. *BnDGAT1-3* and *BnDGAT1-4* were expressed only at low levels throughout seed development, while *BnDGAT1-1* and *BnDGAT1-2* increase in expression 4- to 6-fold through seed development (Fig. 3). While not conclusive, this transcript analysis suggested that BnDGAT1-1 and BnDGAT1-2 are likely to be the most important isozymes for seed TAG synthesis.

To characterize the activity of these enzymes in greater detail, their activity *in vitro* was first analysed using microsome preparations from yeast expressing the individual proteins. Diacyglycerol substrates PO-DAG and OO-DAG were supplied, which are typical of TAG synthesis in *B. napus* seeds, along with 16:0-CoA or 18:1-CoA as the second substrate. In the enzyme specificity experiments, all isozymes incorporated 16:0 into TAG at higher rates than 18:1 (Fig. 5). The ratio of 16:0/18:1 was higher with PO-DAG substrate (average ratio 5.4) than with OO-DAG (average ratio 3.2) (Fig. 4). The ready incorporation of 16:0 in these assays contrasts with the composition of *B. napus* seed oil, in which TAG with 16:0 at the *sn*-3 position amount to only 10% of the total (Neff *et al.*, 1994; Przybylski, 2001). After testing the selectivity of each BnDGAT isozyme using equimolar concentrations of both 16:0-CoA and 18:1-CoA, results substantially mirrored those obtained in the acyl-CoA specificity assays (compare Fig. 6 with Fig. 5) with higher incorporation of 16:0 than 18:1 with either PO-DAG or OO-DAG as cosubstrate. The ratio of 16:0/18:1 incorporated averaged 2.03±0.26 with PO-DAG and 1.81±0.14 with OO-DAG.

During the period of rapid TAG accumulation in *B. napus* seeds from 20 to 40 DAF, the ratio of 16:0-CoA to 18:1-CoA in seed tissue is 1:3 (Perry and Harwood, 1993; Larson and Graham, 2001; Larson *et al.*, 2002). For this reason, a new series of selectivity assays were carried out using 5 µM 16:0-CoA and 15 µM 18:1-CoA. Under these conditions, considerably more 18:1 than 16:0 was incorporated into TAG (Fig. 7). With either PO-DAG or OO-DAG, the ratio of 16:0/18:1 incorporation averaged 0.38±0.04 for the four BnDGAT1 isozymes. This very large change in 16:0/18:1 was mainly caused by a large decrease in 16:0 incorporation. However, this effect is not a result of the lower 16:0-CoA concentration per se, because assays conducted with 16:0-CoA alone at 5 µM (Table 1) gave rates comparable to those for each isozyme in Figs 5 and 6. It is inferred that the changes in incorporation rates in Fig. 7 are the result of the competing 18:1-CoA substrate. The mammalian DGAT1 enzyme has been shown to undergo changes in conformation upon binding of 18:1-CoA (Lopes *et al.*, 2014). If conformational changes also occur in the plant protein, these might reduce the affinity of the enzyme for 16:0-CoA. Although additional biochemical and structural data are needed to understand the mechanism, these results indicate that assays of the four BnDGAT1 isozymes using the ratio 1:3 16:0-CoA/18:1-CoA found in developing *B. napus* seeds can substantially reconcile the enzyme activities with the predominance of 18:1 at the sn-3 position of *B. napus* seed TAG.

*In vivo* analyses of the yeast strains expressing the BnDGAT1 isozymes were used to extend characterization, measuring incorporation of the fatty acids into TAG for cultures grown in media either unsupplemented or supplemented with palmitate or oleate fatty acids. Fatty acids in the TAG fraction of cultures grown without exogenous fatty acid had 16-carbon fatty acids as 45% of the total; the remaining 55% were of the sum of 18:0 and 18:1 (Fig. 8A). When the cultures were supplemented with palmitic acid, 16-carbon fatty acids accounted for ∼55% of the acyl groups in TAG (65% in yeast expressing BnDGAT1-1). By contrast, in cultures supplemented with oleic acid, 16-carbon fatty acids fell to <30% of TAG acyl groups compared to ∼60% as 18:1 (Fig. 8A). Compared to wild-type yeast, each of the H1246 strains expressing a BnDGAT1 isozyme accumulated substantially higher amounts of TAG, with or without supplementation with fatty acid (Fig. 8B). The isozymes exhibiting highest activity in isolated yeast microsomes, BnDGAT1-2 and BnDGAT1-3, accumulated ∼10-fold higher TAG than wild-type, while BnDGAT1-1 and BnDGAT1-4 contained 2- to 4-fold more TAG than wild-type. While palmitate or oleate supplementation strongly altered the fatty acid composition of TAG (Fig. 8A), it had little or no effect on the quantity of TAG accumulated (Fig. 8B).

Phylogenetic analysis (Fig. 1) indicates that there are two pairs of proteins based on homology, BnDGAT1-1 with BnDGAT1-2 and BnDGAT1-3 with BnDGAT1-4. However, the members of these pairs derive from different ancestors of *B. napus*, with BnDGAT1-1 and BnDGAT1-4 descended from the A lineage and BnDGAT1-2 and BnDGAT1-3 descended from the C lineage (Greer *et al.*, 2014). In addition, only one homology pair is highly expressed in *B. napus* developing seeds; the *BnDGAT1-1* and *BnDGAT1-2* genes are expressed a level 3- to 5-fold higher than *BnDGAT1-3* and *BnDAGAT1-4* during the period of TAG accumulation 20 to 40 DAF (Fig. 3). It is surprising that one of the genes highly expressed in developing seeds, *BnDGAT1-1*, encodes an isozyme with low activity in the assays of the heterologously expressed protein. In contrast, the message encoding BnDGAT1-3 is expressed at very low levels, yet codes for an active enzyme when expressed in yeast. It is possible that assays of BnDGAT1-1 and BnDGAT1-3 do not accurately reflect their *in planta* activity. Alternatively, while highly expressed enzymes are typically also highly active, this is not always true when a new species, such as *B. napus*, has recently arisen by merging of two genomes (allopolyploidy) (Chen, 2007). In these cases enzymes may be either active and not highly expressed or highly expressed but not active, as found, for example, in the phytoene synthase family in *B. napus* (Lopez-Emparan *et al.*, 2014).

Understanding the dynamics of fatty acid incorporation into TAG is an important contribution to the goal of manipulating both the composition and quantity of oil in *B. napus*. Recent work with the *Arabidopsis* DGAT2 enzyme has demonstrated some differential incorporation of saturated or unsaturated acyl-CoA substrates (Zhou *et al.*, 2013; Ayme *et al.*, 2014); it may be important for a complete understanding of *B. napus* TAG formation to examine the activity of BnDGAT2 isoforms. The current analysis of substrate specificity and activity of the four BnDGAT1 enzymes will provide guidance in engineering improved quantity and quality of oils from this important oilseed crop.
